# Commentary: The moral bioenhancement of psychopaths

**DOI:** 10.3389/fpsyg.2019.02880

**Published:** 2020-01-08

**Authors:** Elisabetta Sirgiovanni, Mirko Daniel Garasic

**Affiliations:** ^1^Department of Molecular Medicine, Faculty of Pharmacy and Medicine, Sapienza University of Rome, Rome, Italy; ^2^Libera Università Maria SS. Assunta, Rome, Italy

**Keywords:** moral bioenhancement, psychopathy, consent and refusal to treatment, involuntary treatment, open justification

Baccarini and Malatesti (2017) defend the idea that we must use coercively biomedical means to enhance the morality of a specific group of individuals: psychopaths, diagnosed through the Psychopathy Checklist-Revised (PCL-R) standards (Hare, 2003). Their argument is theoretical, thus it goes independently from the actual effectiveness of existent treatments, and it is based on a logical reasoning. Moral bioenhancement (MB) means include psychotropic drugs, brain stimulations, neurosurgeries, genetic editing, etc.

In short, the authors apply Gerald Gaus' account of *open justification* (Gaus, 1996, 2011), according to which “a prescription addressed to an agent is a reasoning that includes premises that consider the system of reasons (such as beliefs, preferences, etc.) of that agent” (Baccarini and Malatesti, 2017, p. 1). In their view, coercive MB of psychopaths is morally sound and deducible by reasons within the psychopath's cognitive-affective system—even if the psychopath needs not to be able to consciously or sincerely endorse them.

Notoriously psychopaths have Machiavellian traits, a dimension in the Dark Triad (Paulhus and Williams, 2002), including anti-sociality and narcissism. In order to exploit others, the psychopath wishes to live in a society where everyone is cooperative except herself. Consequentially, the psychopath would prescribe MB to other psychopaths. The authors state that an agent must apply to herself a prescription she would accept for others, “if she shares with them the relevant characteristics” (i.e., psychopathic traits), and “unless (s)he can justify to others that the two cases are relevantly different” (Baccarini and Malatesti, 2017, p. 3). Since the psychopath possesses the same personality traits of other psychopaths, the authors claim we would be justified, in Kantian terms, to universalize the prescription of mandatory MB to her.

We believe that this argument is flawed. In sum, we argue that the psychopath's cognitive-affective system would consistently justify reasons against mandatory MB to herself, even if she wishes differently for others, and that the prescription cannot be extended. What “immoral rule” is the best deducible from the psychopath's cognitive-affective system? If we think of human morality as cooperation in evolutionary terms (Curry, 2016), as the authors do, it seems that psychopaths contradict what has been held inter-culturally as a guiding principle of reciprocity, the Golden Rule. On the contrary, psychopaths respond to what we may call, from the triad, a Dark Rule. Psychopaths believe and feel that “one can treat others (i.e., manipulating, hurting, torturing, killing, etc.) in ways that one would not like to be treated.” In fact, there is no evidence that psychopaths wish to be treated (even unconsciously) in the same ways they treat others. Research shows that when viewing stimuli depicting bodily injuries adopting an image-self perspective, psychopaths have normal neural responses for pain (Decety et al., 2013). These responses do not match the atypical patterns of brain activation psychopaths show when adopting an other-perspective. Thus, the psychopath can consistently justify within her cognitive-affective system that her own case and the other psychopaths' case are relevantly different.

It could be objected that a Dark Rule entails for the psychopaths to accept to be treated by others in ways they do not like to be treated. Yet, we should keep in mind that, for a Kantian, the Dark Rule (i.e., treating others as a means) is intrinsically unethical, hence it is *not* a universalizable rule.

Having pointed out this unconvincing dimension of Baccarini and Malatesti's account, we wish to next raise objections about forcing MB on psychopaths even if that was indeed the case.

Involuntary treatment has been justified by combining public reasons of social security (Persson and Savulescu, 2012, 2019) with other criteria implemented in different legislations (Saya et al., 2019), such as mental incapacity and non-intrusiveness of the treatment. Remarkably, all these criteria are now challenged by recent international standards for the rights of persons with disabilities, where informed consent to mental health services has been vigorously supported in any case (see United Nations, 2006, art. 14; United Nations, 2008, par. 64–65; United Nations, 2019).

With regard to MB of psychopaths, it is questionable that these criteria can be met.

In most cases, it is doubtful to claim that the psychopath's volition is harmed. Remarkably, psychopaths are multifaceted in decision-making, by mainly lacking emotional engagement in moral choice/action while their rational judgment is unimpaired (Cima et al., 2010; Aharoni et al., 2014; Jurjako and Malatesti, 2016). Evolutionists do not see psychopathic traits as expression of an underlying dysfunction, but as a persisting adaptation to certain environments (Glenn et al., 2011). Notably, there are still discrepancies between the PCL-R construct of psychopathy and the corresponding official category of antisocial personality disorder (ASPD) in the DSM (Few et al., 2015). These considerations together could reinforce the argument that we are not totally entitled to classify psychopathy as a proper mental incapacitation. It must be noticed that PCL-R diagnoses are over-inclusive, since the scale attributes psychopathic traits dimensionally to a large group of people, including non-offending and subclinical individuals such as businessmen, lawyers, actors, politicians, and rebels of various sort, not only serial killers and recidivist offenders (Skeem et al., 2011).

Most importantly, MB is far from being the least restrictive or intrusive treatment. This might exclude most MB means, especially those that are irreversible (e.g., neurosurgeries), impact severely on intertwined functions (e.g., psychotropic drugs, brain stimulations, etc.), and that pass on through generations unpredictably (i.e., gene editing).

Moreover, the call for involuntary treatment is not as neutral and objective as often depicted by its promoters (Garasic, 2013). The “greater good for society” behind the suspension of human rights is often charged with biopolitical values, and it exploits the patient/prisoner as a tool to reinforce or instill specific norms/standards in the society. The defense of coercive MB hides an idea of “moral perfectionism” (Cavell, 1990), according to which we must conform to an idealistic and demanding account of morality where moral imperfections or differences are never tolerated and need to be eliminated. Defining the “morally perfect” is a challenge as much as concluding that a society without moral defects would be a better society. What is the prototypical “moral individual” into whom we should transform the psychopath?

This approach creates substantial frictions with the individual rights. For its *moral* specificity, coercive MB interferes tremendously with individual autonomy and freedom without empowering moral competence (Harris, 2011, 2016; Corbellini and Sirgiovanni, 2015). Personal preferences/options belong to a larger spectrum of moral acceptability than that conventionally prescribed by society in a given historical time.

Furthermore, it is unclear whether we should prescribe mandatory MB also to non-psychopathic offenders and preventively to non-offending or subclinical psychopaths. The same reasons of social security, in fact, seem to predispose ourselves (and society) to large extensions of the legitimacy of MB.

In conclusion, we defend the view that the right to refuse MB must be protected. It seems that without consent, psychopathic offenders' incarceration or admission to psychiatric facility are still the only acceptable security measures.

## Author Contributions

The authors discussed, reviewed, and approved together the entire manuscript. ES conceived and wrote the first part of the manuscript and half of the second part, MG conceived and wrote the second part.

### Conflict of Interest

The authors declare that the research was conducted in the absence of any commercial or financial relationships that could be construed as a potential conflict of interest.
